# Tension Pneumocephalus With Acute Cerebellar Symptoms Due to an Intradiploic Epidermoid Cyst Eroding the Mastoid Bone

**DOI:** 10.7759/cureus.60427

**Published:** 2024-05-16

**Authors:** Jarne Schepens, Frederic Van Havenbergh, Joost Dejaegher, Philippe Demaerel, Raf Sciot, Steven De Vleeschouwer

**Affiliations:** 1 Department of Neurosurgery, University Hospitals Leuven, Leuven, BEL; 2 Department of Neurosurgery, ZAS Hospitals, Antwerp, BEL; 3 Department of Radiology, University Hospitals Leuven, Leuven, BEL; 4 Department of Imaging and Pathology, University Hospitals Leuven, Leuven, BEL; 5 Department of Neurosciences, KU Leuven, Leuven, BEL

**Keywords:** bony erosion, ball-valve effect, mastoid bone, tension pneumocephalus, intradiploic epidermoid cyst

## Abstract

This case report presents a unique presentation of an intradiploic epidermoid cyst (IDEC) in a 55-year-old female. She presented with acute cerebellar symptoms triggered by a Valsalva maneuver. IDECs are a rare type of intracranial epidermoid cysts. They are benign and have a slow growth pattern that translates into progressively developing symptoms instead of acute symptoms. Symptoms include local deformities, focal neurologic deficits, and pain. This patient developed acute cerebellar symptoms due to erosion of the mastoid bone that created a pathway between the eustachian tube and the intracranial space via the mastoid air cells. Consequently, tension pneumocephalus emerged via a ball-valve effect that caused a significant mass effect in the posterior fossa. Surgical resection of the IDEC and closing of the mastoid air cells resulted in symptom relief by restoring the integrity of the intracranial-extracranial barrier. This case highlights that a higher level of vigilance is warranted for an IDEC in the proximity of aerated bone structures, such as the mastoid air cells and the paranasal sinuses, and that a more proactive approach is advocated.

## Introduction

Intradiploic extradural epidermoid cysts (IDECs) are a rare type of intracranial epidermoid cysts. Epidermoid cysts are a benign tumor (WHO grade I) that account for 0.2 - 1.8% of the intracranial tumors [1]. Epidermoid cysts are divided into intradural epidermoid cysts and extradural epidermoid cysts. The intradiploic extradural epidermoid cysts account for 25% of all epidermoid cysts [1]. It is considered as a congenital lesion that develops from the entrapment of ectodermal remnants in the diploic space of the cranial vault during the third to fifth weeks of gestation. In rare cases, a penetrating trauma might be the underlying etiology by introducing epithelial cells in the diploic space. This results in a sequestration of ectodermal remnants within the intradiploic space [2,3]. The most frequent location are the occipital, the frontal, and the parietal bone [1,4].

It has an indolent nature with a slow growth pattern resulting from the accumulation of dividing epidermal cells [4]. This progressive growth may result in the destruction of the internal table and the thinning of the external table of the skull. As a result, symptoms usually present gradually due to the local mass effect. It causes a variety of symptoms, such as deformations due to displaced soft tissue, pain, tenderness, focal neurologic deficits, or intracranial hypertension in rare cases [1,5,6]. The most common symptom is the local mass effect on soft tissues that causes local deformities [1].

In our case, the patient presented with acute-onset cerebellar symptoms caused by tension pneumocephalus. The IDEC eroded the mastoid bone and this resulted in tension pneumocephalus. The intracranial air caused a significant mass effect on the cerebellar hemisphere and the brainstem.

## Case presentation

Case description

A 55-year-old female presented with progressive headache, nausea, and vomitus. She developed her symptoms after a Valsalva maneuver (coughing). She had a history of a transient ischemic attack (TIA) 11 years earlier. During the work-up for her TIA, a left-sided extra-axial lesion was detected coincidentally in the posterior fossa. Based on the radiographic findings, it was most compatible with an epidermoid cyst. During the first three years of follow-up, the lesion remained stable. Subsequently, she was lost to follow-up.

A neurologic exam at presentation showed a somnolent (Glasgow coma scale of 14) patient with left-sided ataxia. She was transferred to our department for further investigations, monitoring, and treatment. Surgical treatment was performed within 48 hours.

Investigations

**Figure 1:**
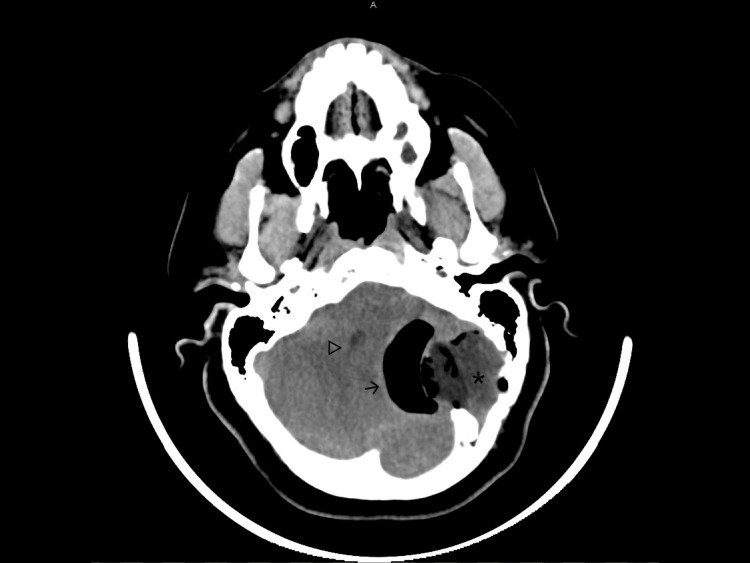
Preoperative CT scan showing a lesion (asterisk) originating from the intradiploic space and surrounded by intracranial air (arrow). It results in mass effect on the cerebellar hemisphere and compression of the fourth ventricle (triangle).

**Figure 2:**
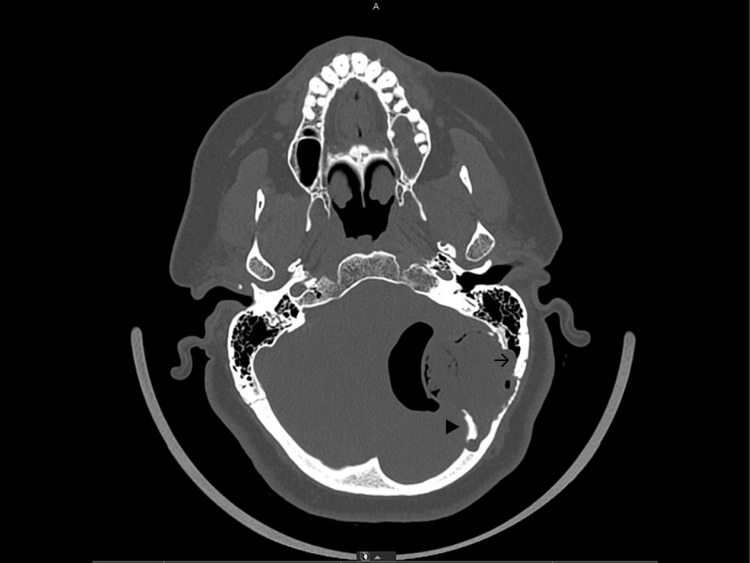
Preoperative CT scan showing erosion of the anteromedial part of the mastoid air cells (arrow) and inward displacement of the internal table (triangle).

A CT scan showed a left-sided mass in the posterior fossa originating from the intradiploic space of the occipital bone with an erosion of the internal and external table of the skull. There was an erosion of the anteromedial part of the mastoid bone. Part of the internal table was displaced inwards due to the growth of the lesion. The most remarkable finding was the intracranial air surrounding the lesion. The fourth ventricle was compressed which resulted in an enlargement of the supratentorial ventricular system (Figure 1, 2).

**Figure 3:**
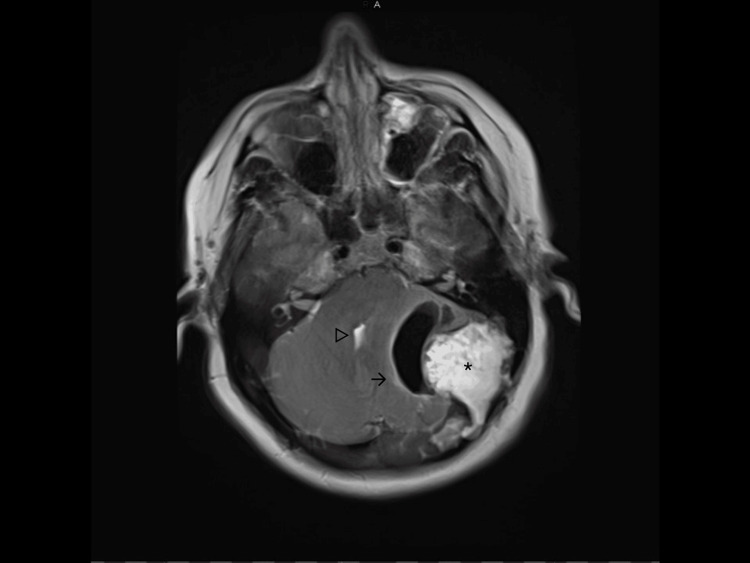
Preoperative MRI-T2 sequence showing a T2 hyperintense (asterisk) lesion surrounded with intracranial air (arrow) and mass effect compressing the fourth ventricle (triangle). MRI: Magnetic resonance imaging

**Figure 4:**
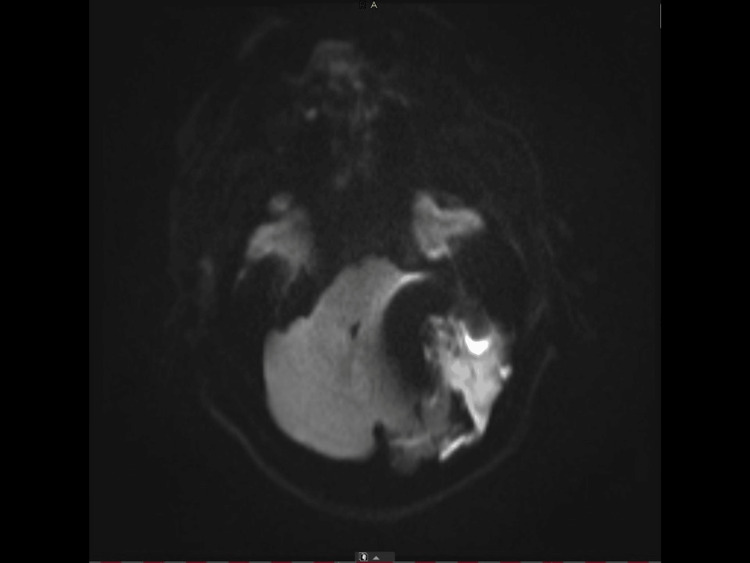
Preoperative MRI-DWI sequence showing diffusion restriction of the lesion content that was highly suggestive of an epidermoid cyst. MRI-DWI: Magnetic resonance imaging-Diffusion-weighted imaging

**Figure 5:**
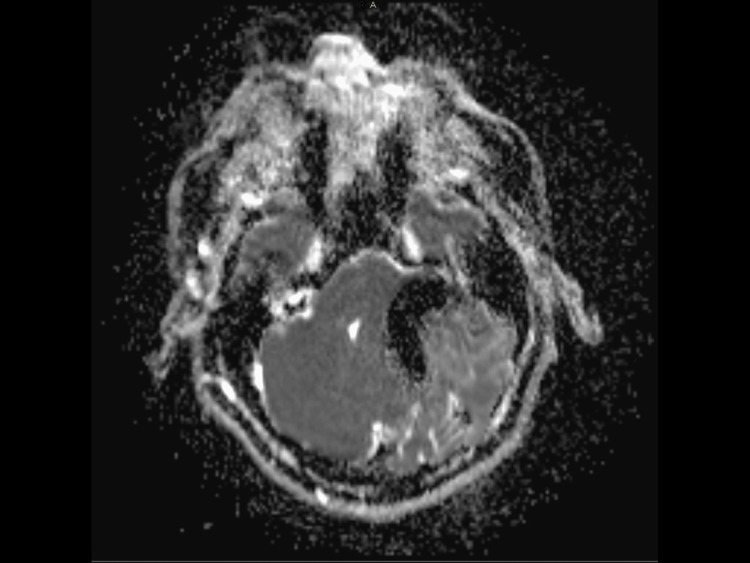
Preoperative MRI-ADC sequence showing diffusion restriction of the lesion content that was highly suggestive of an epidermoid cyst. MRI-ADC: Magnetic resonance imaging-Apparent diffusion coefficient

An MRI confirmed the presence of a mass with surrounding air on the left side in the posterior fossa. The diffusion restriction of the lesion content was highly suggestive of an epidermoid cyst. There was a minimal increase in volume in comparison with the CT scan of 2014. There was a significant mass effect on the cerebellar hemisphere, the pons, and a caudal shift of the cerebellar tonsils. The fourth ventricle was compressed resulting in a slight enlargement of the supratentorial ventricular system in comparison with her previous CT scan. There was an erosion of the surrounding occipital and mastoid bone with adjacent intracranial air. The transverse and sigmoid sinuses on the left side were not visible and suspected to be compressed or invaded (Figure 3-5).

Treatment

A surgical resection was performed to achieve a decompression and a sealing of the mastoid was performed to prevent further accumulation of intracranial air or future cerebrospinal fluid (CSF) leakage. We performed a two-step surgery during the same procedure: first, a right-sided external ventricular drain (EVD) via Kocher’s point was placed in the supine position. A prophylactic EVD was placed as a safety measure to obtain direct access to CSF drainage during the procedure and to treat secondary hydrocephalus in the early postoperative phase. Second, we performed a modified lateral suboccipital approach in the right-sided lateral decubitus. We used a hockey stick incision with an extension to C2. When reaching the occipital bone, we decided to drill the bone instead of performing a craniotomy because of the extensive erosion of the occipital bone. Next, we reached the lesion which was macroscopically most compatible with an epidermoid cyst (pearl-like appearance). Following this, the capsule of the epidermoid cyst was identified and dissected from the underlying dura mater. We could not create a safe dissection plane between the capsule and the transverse and sigmoid sinus due to the adherence and risk of tearing the sinuses. Therefore, we did not remove the capsule overlying the sinuses. On inspection, the dura mater was no longer intact in several places due to invasion of the lesion and also due to involuntary opening of the dura during removal of the capsule. We decided to open the dura to inspect the cerebellum and remove any intradural tumor components. Intradurally, no tumor was visible. Next, the dura mater was reconstructed and closed primarily and sealed with Tachosil. We sealed the open mastoid air cells with bone wax, Tachosil, and Tisseel. Abdominal fat was harvested and placed in the epidural space to fill up the death space. Finally, we used a titanium mesh to reconstruct the bone defect.

Histopathology

**Figure 6:**
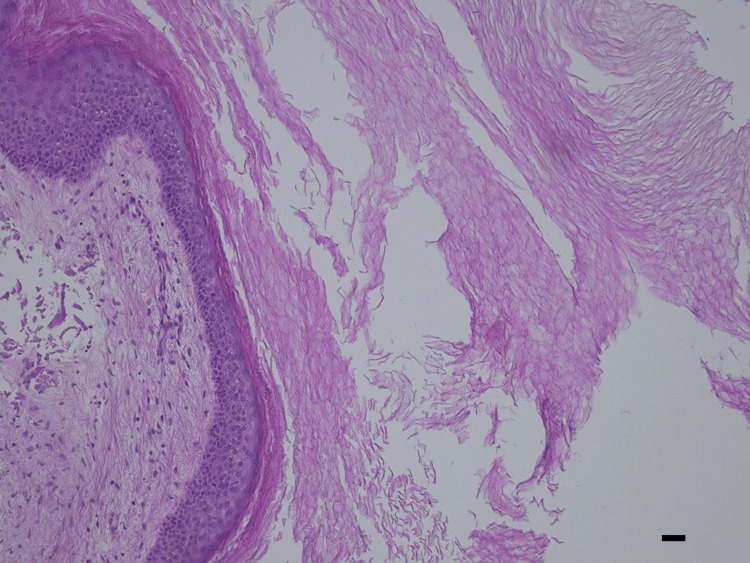
Hematoxylin & eosin staining. The cyst is delineated by a keratinizing multilayered squamous epithelium (original magnification x400, bar=20micron)

The findings of the pathologic report were consistent with an epidermoid cyst. Decalcification of the bone showed keratinized material and a few fragments delineated by a multilayered squamous epithelium with no atypia and no adnexa (Figure 6).

Outcome and follow-up

**Figure 7:**
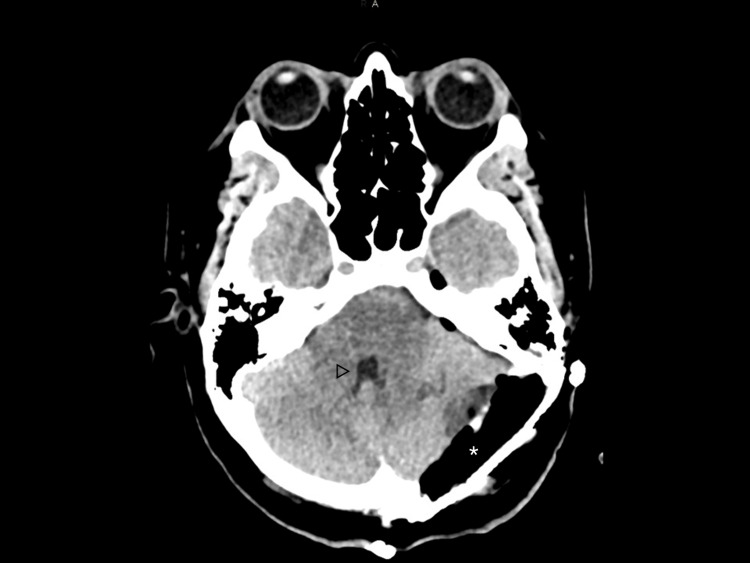
Postoperative CT showing resection of the lesion. Fat graft (asterisk) in the epidural space and opening of the fourth ventricle (triangle).

**Figure 8:**
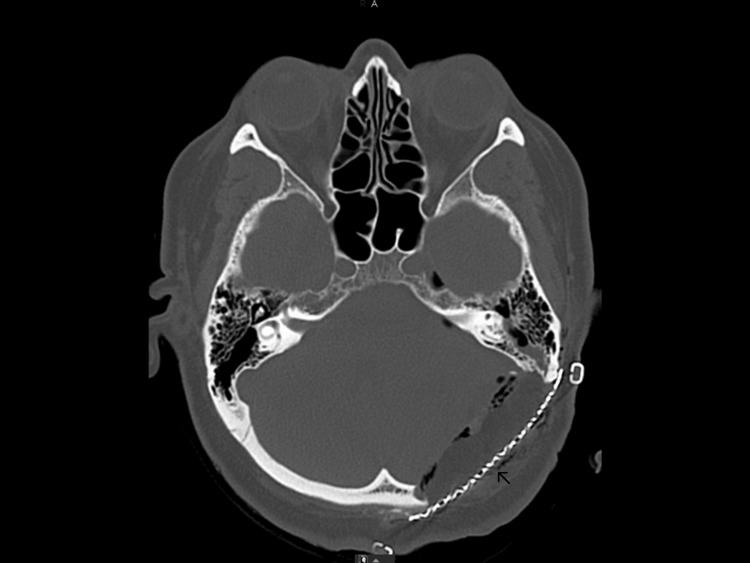
Postoperative CT scan showing reconstruction with a titatinum mesh (arrow).

During the early postoperative phase, we noticed a slow recovery of the cerebellar symptoms with recuperation of the left-sided ataxia. She was admitted to the ICU for 1 day and was subsequently hospitalized for 5 more days. The external ventricular drain was kept closed and was removed after 3 days. A postoperative CT scan showed no postoperative complications (Figure 7, 8). A CT scan 48 hours after removal of the EVD showed no signs of hydrocephalus.

**Figure 9:**
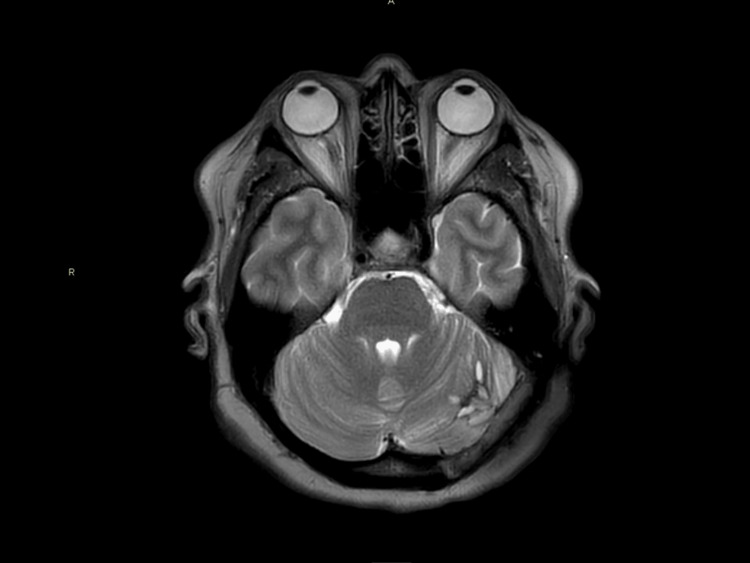
Postoperative MRI: T2 sequence showing no residual tumor and disappearance of the mass effect. MRI: Magnetic resonance imaging

During the late postoperative phase 6 months postoperatively, we noticed no residual neurologic deficit. MRI 6 months postoperatively showed no residual tumor (Figure 9).

## Discussion

To our knowledge, our case report is the first to illustrate an intradiploic extradural epidermoid cyst presenting with acute-onset cerebellar symptoms due to tension pneumocephalus. A recent case report illustrated a case of pneumocephalus due to erosion of the frontal sinus [7]. Two other case reports have also demonstrated the risk of tension pneumocephalus due to erosion of the frontal sinus [8,9]. In our patient, the lesion eroded the mastoid bone resulting in a connection between the mastoid air cells and the intracranial cavity which forms a pathway between the intracranial cavity and the eustachian tube. This growth pattern, erosion of the internal table, and thinning of the external table are typically seen in IDEC cases [3].

The acute-onset symptoms were triggered by a Valsalva maneuver and not by a rapid increase in volume. Two mechanisms are hypothesized for spontaneous pneumocephalus: (1) a ball-valve effect and (2) an inverted-soda-bottle effect. The ball-valve effect is based on the unidirectional movement of air to intracranial due to a positive pressure gradient. The positive pressure gradient might be created by a Valsalva maneuver. This mechanism attributes a valve-like function to the Eustachian tube. The inverted-soda-bottle effect is based on a negative pressure gradient due to CSF leakage resulting in the aspiration of air. Both mechanisms require a connection between the intra - and extracranial space. The connection might be a consequence of a bony defect due to trauma, a congenital defect, hyperpneumatization, or destruction by an invasive lesion [10]. In this case, we believe the ball-valve effect resulted in the tension pneumocephalus as the symptoms were triggered by a Valsalva maneuver. Erosion of the mastoid air cells, due to the epidermoid cyst, resulted in a trajectory from the intracranial space to the mastoid air cells, the attic, the tympanic cavity, and finally the eustachian tube [11].

Symptoms typically develop gradually instead of suddenly and depend on the location. Clinical manifestations include local deformities (subcutaneous lump), tenderness, headache, and neurologic symptoms (seizure, focal neurologic deficit, meningitis) [5,6]. Some cases have been reported with intra-orbital expansion causing exophthalmos or diplopia [3]. IDECs also present rarely with intracranial hypertension, either primarily due to a significant mass effect or secondary due to venous occlusion [12].

The treatment consists of surgical resection including the complete cystic wall to prevent recurrence [2,3]. A consensus regarding the extent of resection of adjacent bone structures is lacking [3]. In our patient, the cystic wall resection was incomplete due to its adherence to the sigmoid and transverse sinus. In our case, we decided to place a prophylactic EVD. The indication for prophylactic EVD placement to treat secondary, postoperative hydrocephalus in fossa posterior lesions lacks consensus. The patient had an enlargement of the supratentorial ventricular system due to compression of the fourth ventricle. A recent study identified preoperative hydrocephalus and perilesional edema as independent risk factors for postoperative CSF drainage. The rate of shunt-depended patients at 3-month follow-up was 10.1% [13].

## Conclusions

An intradiploic epidermoid cyst is a slow-growing tumor that usually presents with gradual onset symptoms. However, acute-onset symptoms are possible. In our case, it was due to bony erosion of the mastoid resulting in tension pneumocephalus. The mechanism is based on a ball-valve effect, where a Valsalva maneuver creates a positive pressure gradient forcing air from the mastoid to the intracranial cavity. It also illustrates that a higher level of vigilance is warranted for intradiploic epidermoid cysts in the proximity of aerated bone structures, such as the mastoid air cells and the paranasal sinuses, and that a lower threshold for surgery might avoid more complex reconstructive surgery in urgent circumstances.
